# Water Complexes of Cytochrome P450: Insights from Energy Decomposition Analysis

**DOI:** 10.3390/molecules18066782

**Published:** 2013-06-10

**Authors:** Nandun Thellamurege, Hajime Hirao

**Affiliations:** Division of Chemistry and Biological Chemistry, School of Physical and Mathematical Sciences Nanyang Technological University, 21 Nanyang Link, Singapore 637371, Singapore

**Keywords:** cytochrome P450, EDA, LMOEDA, resting state, compound I, water

## Abstract

Water is a small molecule that nevertheless perturbs, sometimes significantly, the electronic properties of an enzyme’s active site. In this study, interactions of a water molecule with the ferric heme and the compound I (Cpd I) intermediate of cytochrome P450 are studied. Energy decomposition analysis (EDA) schemes are used to investigate the physical origins of these interactions. Localized molecular orbital EDA (LMOEDA) implemented in the quantum chemistry software GAMESS and the EDA method implemented in the ADF quantum chemistry program are used. EDA reveals that the electrostatic and polarization effects act as the major driving force in both of these interactions. The hydrogen bonding in the Cpd I•••H_2_O complex is similar to that in the water dimer; however, the relative importance of the electrostatic effect is somewhat larger in the water dimer.

## 1. Introduction

The ubiquitous heme-containing monooxygenase, cytochrome P450 (P450), has been extensively studied both experimentally and computationally, because of the important role it plays in oxygenating organic compounds [1,2,3,4]. The active site of the enzyme contains an iron atom bound to a protoporphyrin IX ligand and two other “proximal” and “distal” ligands. The proximal ligand is a thiolate from a cysteine residue of the protein, whereas the distal ligand is variable and changes during the catalytic cycle.

**Figure 1:**
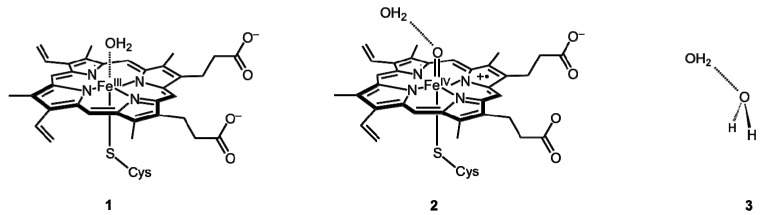
Resting state (**1**), water-bound Cpd I (**2**), and water dimer (**3**).

In the so-called “resting state” (**1** in Figure 1), the distal ligand is a H_2_O molecule, which is displaced when a substrate enters. The iron atom of the resting state is in its ferric Fe^3+^ state. Compound I (Cpd I) is an oxoiron(IV) porphyrin π-cation radical (**2** in Figure 1), and is believed to be responsible for the monooxygenase activity of P450s [5,6]. In this case, the distal ligand is an oxo group that is transferred to the substrate during the reaction, and thereafter, the oxygenated product is released from the active site to restore P450 to the resting state. There has been much work done to study the hydrogen abstraction from camphor by P450cam Cpd I, using QM/MM methods whereby the protein environmental effect was taken into account [7,8,9,10]. It has been noted that a H_2_O molecule interacting with the oxo ligand of Cpd I lowers the activation barrier for the H-abstraction step, and that this interaction is mainly of electrostatic origin [10,11]. Also, this interaction has been calculated using a QM/MM method [12].

Energy decomposition analysis (EDA) is a very helpful tool to dissect the physical origins of intra- or intermolecular interactions [13,14,15,16,17,18,19,20,21,22,23,24]. In the past several decades, a number of EDA methods have been proposed. For example, Su *et al*. developed a method called localized molecular orbital energy decomposition analysis (LMOEDA) and demonstrated its applicability to various interacting systems [14]. In this method, the total interaction energy is decomposed into electrostatic, exchange, repulsion, polarization and dispersion components. Another widely used EDA method is the one implemented in the ADF program [19,20]. In this EDA, the total interaction energy is decomposed into electrostatic, Pauli repulsion, and orbital interaction terms. We used the LMOEDA and ADF-EDA methods in this study because they can be applied to interactions between molecules having open-shell electronic structures. 

Usually in EDA methods, the total interaction energy is defined as the difference between the energy of the supermolecule and the energy of the monomers (or fragments) [13]. This interaction energy is then partitioned into a few terms. The electrostatic energy term can be defined as the interaction between the static charge densities of each monomer within the supermolecule. This term includes the attractive Coulomb interactions between nuclei of one monomer with the electrons of the other monomer, repulsive Coulomb interactions between the nuclei of each monomer and the repulsive Coulomb interactions between the electrons of each monomer. The total electrostatic interaction is normally attractive. The Pauli term contains the exchange and repulsion energies, which are stabilizing and destabilizing, respectively. The exchange interaction arises due to the antisymmetric nature of a wave function that allows electrons to exchange between monomers. The repulsion interaction originates largely from other types of 2-electron integral terms for monomer orbitals. In the LMOEDA method these two effects are separated [14], whereas in the Kitaura-Morokuma method [18] and several other methods [19,20,21,22,23,24] these may be combined. In the latter treatment, the Pauli energy is repulsive. Polarization is a stabilizing effect that is caused by relaxation of the supermolecular wave function. This is also known as the orbital relaxation energy and is always attractive. In some methods, this is known as orbital interaction energy [19,20]. This term is a major indicator of the covalent nature of a bonding interaction [13,14]. In the two methods that we employ here, both of the intramolecular polarization and intermolecular charge-transfer contributions, in the context of the Kitaura-Morokuma scheme, are included in this term. The “dispersion” term computed in the LMOEDA method is an attractive term, which arises due to electron correlation [14]. When the EDA relies on DFT energies, this term is defined as the difference in the correlation energy between the supermolecule and those of monomers. Thus, this term depends on the quality of the correlation functional used for the calculation.

EDA should be useful in understanding the origins of the complex environmental effects of metalloenzymes on the active site [12,25]. In this study, we focus on the interaction of H_2_O with the ferric heme unit in **1** and Cpd I in **2**. H_2_O is a small molecule but sometimes exerts a significant influence on the active-site properties such as reactivity. EDA methods are used to gain an understanding of the physical origins of these interactions. For comparison, the water dimer **3** (Figure 1) is also considered.

## 2. Results and Discussion

### 2.1. LMOEDA of Complexes **1**–**3**

**Figure 2:**
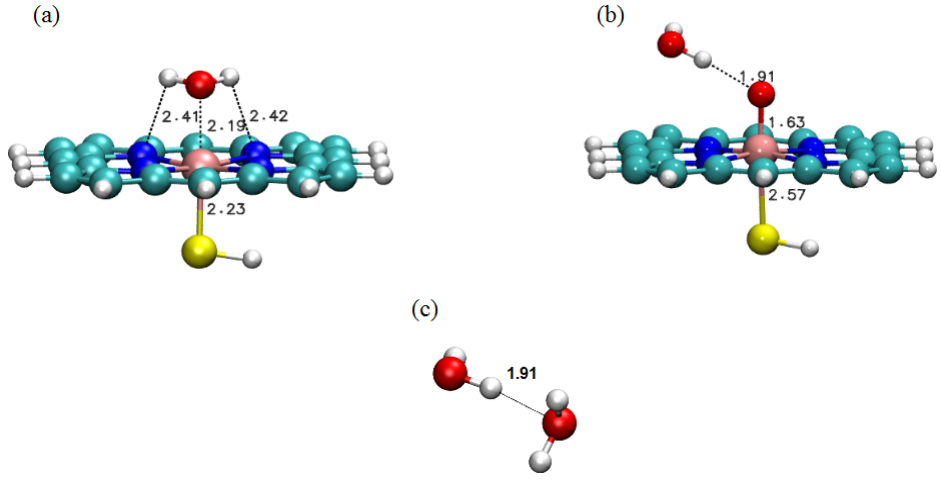
Optimized geometries of (**a**) **1**, (**b**) **2**, and (**c**) **3**. Distances are in Å.

The optimized structure of **1** is depicted in Figure 2a. The orientation of the water molecule can be upright or tilted [26,27]. In the current study, the tilted form of water is observed. This form is stabilized by two H bonds, which are formed between water H atoms and two porphine N atoms. The geometrical parameters are similar to those reported previously [26].

LMOEDA-decomposed energy terms for the interaction between H_2_O and the other fragment in **1**–**3** are summarized in Table 1. In complex **1**, the strongest attractive interaction is the electrostatic interaction (−33.30 kcal/mol), followed by the exchange, polarization and dispersion interactions. The relatively large electrostatic energy is understandable because the water oxygen takes on some negative charge and the iron ion is formally cationic (Fe^3+^). The exchange stabilization is also large, −22.30 kcal/mol, but the destabilization due to repulsion is much larger (+72.40 kcal/mol). The molecular orbitals of a monomer will change when there is another monomer in its vicinity. Furthermore, there will be orbital interactions between monomers that permit charge transfer. These effects are accounted for by the polarization interaction. In the case of **1**, this effect amounts to −21.96 kcal/mol. The relatively large polarization effect should be attributed, to a large extent, to the charge transfer from the water lone-pair orbital to the vacant d_z2_-type orbital of ferric heme. The dispersion interaction made the smallest, yet non-negligible contribution (−8.91 kcal/mol) to the total interaction energy.

**Table 1:** LMOEDA decomposed energy terms in kcal/mol.

	1	2	3
Electrostatic	−33.30	−8.34	−8.97
Exchange	−22.30	−2.67	−3.01
Repulsion	+72.40	+14.12	+12.39
Polarization	−21.96	−7.99	−5.53
Dispersion	−8.91	−3.54	−2.58
Total	−14.07	−8.41	−7.70

The optimized geometry of **2** is presented in Figure 2b. The geometry is similar to that obtained by Kumar *et al*. using the LACVP/6-31G basis set [11]. The O•••H distance reported in their study was 1.84 Å, whereas it is 1.91 Å in the current study. Of all the attractive interactions, the strongest ones are the electrostatic and polarization interactions. 

The total interaction energy of **2** is smaller by ~6 kcal/mol than that of **1**. This is because the interaction between the H_2_O and Cpd I is described as a H bond, which is not as strong as a metal-ligand bond, as in **1**.

Altun *et al*. observed in their QM/MM study that the activation barrier of the H abstraction reaction of P450cam is lowered by 4 kcal/mol because of favorable electrostatic interactions between the oxo ligand and a nearby water molecule, w903 [10]. This observation was further confirmed by a series of DFT calculations carried out by Kumar *et al*. [11]. The latter study observed that the strength of the hydrogen bond between the water molecule and the oxo atom increases during the hydrogen abstraction because the oxo atom becomes more negatively charged. The LMOEDA decomposed energy terms show that the electrostatic interaction is indeed a driving force of this interaction between Cpd I and H_2_O. Besides, polarization also appears to contribute to the attractive interaction nearly to the same extent (Table 1).

The decomposed energy terms of **2** and **3** can be compared to understand the difference in the nature of H bonds. In either case, the H•••O distances are the same, and the total interaction energy is similar. However, a noticeable difference can be seen in the relative importance of the electrostatic and polarization terms. As we have seen earlier, **2** has nearly the same contributions from these two interaction terms. In contrast, **3** exhibits a larger weight of the electrostatic term, and the polarization effect is smaller, consistent with previous results [18,22,23]. If the relative contribution to the attractive interaction is considered, in **2**, it is 37% for electrostatic, 35% for polarization, 11% for exchange and 15% for dispersion. In **3**, it is 45% for electrostatic, 28% for polarization, 15% for exchange and 13% for dispersion. This clearly shows that the polarization is more important in **2** than in **3**.

### 2.2. ADF-EDA of Complexes **1** and **3**

The Cpd I fragment in **2** in the doublet spin state has a triradicaloid character [1]. Description of this electronic state by DFT requires the use of the unrestricted formalism, and thus may not adequately be done by ADF-EDA. Therefore, ADF-EDA was performed only for **1** and **3**. ADF-EDA decomposed energy terms are summarized in Table 2. Despite the difference in the level of theory employed for calculations, the total interaction energies obtained by LMOEDA and ADF-EDA are more or less similar to each other. The Pauli repulsion term calculated by ADF-EDA is similar to the sum of exchange and repulsion terms of LMOEDA. According to both EDA schemes, the main driving force of this interaction in **1** is the electrostatic interaction, and the polarization or orbital-interaction stabilization is smaller.

**Table 2:** ADF-EDA decomposed energy terms in kcal/mol.

	1	3
Electrostatic	−40.28	−9.14
Pauli repulsion	+50.86	+8.59
Orbital interaction	−24.96	−4.21
Total	−14.38	−4.75

### 2.3. Spin and Charge Distributions

Table 3 presents the Mulliken charge and spin distributions on various subgroups in the isolated and complex forms of **1**, as obtained using GAMESS. The attractive charge-charge interactions occurring between Fe and O as well as between H and N are the major origins of the electrostatic interaction. In the H_2_O molecule, the magnitude of charge on the H atoms increases by ~0.05 e upon complexation, whereas the magnitude of the charge of the O atom decreases by 0.07 e. Overall, the total charge of H_2_O increased by +0.18 e in the complex form, whereas the ferric heme fragment accepted the same amount of negative charge. This indicates that there was charge transfer from the water to the ferric heme. Indeed, the positive charge of the Fe group decreased by 0.10 e upon complexation and the magnitude of the negative charge on the porphine group has increased by approximately the same amount. 

Table 3 also shows how the spin population of **1** has changed upon complexation. The spin populations on the Fe atom and the porphine groups decrease slightly and the population on the SH group changes the sign while decreasing its magnitude. These changes in partial charge and spin are reflected in the polarization term of the LMOEDA scheme.

**Table 3:** Charges and spin populations of various groups in the fragments and complex of **1**.

Group	Charge		Spin	
	In fragments	In complex	In fragments	In complex
H_a_	+0.39	+0.45	0.00	0.00
O	−0.77	−0.70	0.00	0.00
H_b_	+0.39	+0.44	0.00	0.00
Fe	+0.50	+0.40	+1.25	+1.04
Por	−0.48	−0.59	−0.14	−0.07
SH	−0.02	+0.01	−0.11	+0.03

Charge distributions and spin populations of various subgroups of H_2_O and Cpd I in **2** in their isolated and complex forms, as obtained with GAMESS, are summarized in Table 4. Upon complexation, the originally symmetrical charge distribution of H_2_O is changed. The charge on the H atom, which is closer to the oxo group (H_a_) undergoes a larger change (from +0.38 to +0.45) than that of the other hydrogen atom (H_b_, from +0.39 to +0.38). The charge on the oxo group is −0.40 in the isolated state. In the case of **1**, the charges of Fe and the water oxygen were +0.50 and −0.77, respectively (Table 3), in the isolated state. Apparently, the difference in charges of the interacting pair in **1** is larger than in **2**, which is reflected by the larger electrostatic energy in **1**. Interestingly, the charge of the whole water moiety does not change in **2**, which is different from the case of the water dimer, as we shall see later. The partial charge is changed after complexation only on oxo and porphine groups of Cpd I. Compared to **1**, the overall change of charge upon complexation is smaller in **2**. This is reflected in a weaker polarization interaction in **2** than in **1**. After complexation, the magnitudes of spin populations on Fe and porphine have slightly increased while they are slightly decreased on the oxo and SH groups. However, overall, the triradicaloid state is maintained even after complexation. 

**Table 4:** Charges and spin populations of various groups in the fragments and complex of **2**.

Group	Charge		Spin population	
	In fragments	In complex	In fragments	In complex
H_a_	+0.38	+0.45	0.00	0.00
O	−0.77	−0.83	0.00	+0.01
H_b_	+0.39	+0.38	0.00	0.00
Fe	+0.70	+0.70	+1.18	+1.28
Por	−0.23	−0.17	−0.51	−0.55
Oxo	−0.40	−0.48	+0.90	+0.81
SH	−0.06	−0.06	−0.54	−0.55

In **1**, there are three significant interactions between partial charges, that is, the interaction between partial charges on Fe and water O atoms and the two interactions between the partial charges on porphine N and water H atoms. However, in **2**, the only significant interaction between the partial charges is the one between H_a_ and the oxo group. Since there are a larger number of closer partial charge interactions in **1** than in **2**, and also both O and H_b_ in water are more than 3.0 Å away from the atoms of Cpd I, there is a stronger electrostatic interaction in **1** than in **2** as seen in Table 1.

The charge distribution on the atoms of water molecules in their isolated and dimer forms are presented in Table 5. This is compared with the partial charges of **2**. In the water dimer, the first water molecule has a total charge of +0.07 while the second water molecule has a total charge of −0.07. This indicates that the charge is transferred from the lone-pair orbital of H-bond acceptor to the σ*(O–H) orbital of the H-bond donor [23]. In contrast, the water molecule in the complex form of **2** still has a zero charge, which indicates that there is no overall charge transfer between monomers. This might be because there is a larger degree of back-donation from the O–H bond to the H-bond acceptor (oxoiron unit) in **2** than in the H-bond of **3**.

**Table 5:** Charges of atoms in the fragments and in complex of **3**.

Group	Charge	
O	−0.77	−0.75
H_a1_	+0.39	+0.41
H_a2_	+0.39	+0.41
O	−0.77	−0.84
H_b1_	+0.39	+0.40
H_b2_	+0.39	+0.37

## 3. Computational Methods

The structures presented in Figure 1 are used as models of **1** and **2**, respectively. These structures were optimized with the UB3LYP method using the LANL2DZ effective core potential basis set for iron atom and the 6-31G* basis set for the other atoms. All geometry optimization calculations were done using Gaussian 09 [28]. EDA was performed on these optimized structures with the LMOEDA [13] method implemented in GAMESS [29,30] and the EDA method [19,20] implemented in ADF [31,32]. The UB3LYP/[LANL2DZ, 6-31G*] method was used in LMOEDA calculations. It should be noted that the meaning of UB3LYP/[LANL2DZ, 6-31G*] in GAMESS is slightly different from that in Gaussian 09. For ADF-EDA, the UB3LYP/TZP level of theory was used with out any frozen cores. The basis set superposition error (BSSE) was not corrected in the EDA calculations as it may give rise to SCF convergence problems in DFT methods [14].

In **1**, Fe, porphine and SH were considered as one monomer for LMOEDA (or a fragment for ADF) and H_2_O was considered as the other. Monomer 1 (fragment 1) and the supermolecule had doublet spin multiplicities. In **2**, Cpd I and H_2_O were considered as two monomers for LMOEDA. Again, both monomers are uncharged and Cpd I and the supermolecule had a doublet spin multiplicities. The diagrams of the optimized structures were drawn with visual molecular dynamics (VMD) software [33]. 

## 4. Conclusions

The interactions of two water complexes of P450 in the catalytic cycle were analyzed using the LMOEDA and ADF-EDA methods. From the results obtained by these analyses, it can be concluded that the main driving force of the interaction in the resting state is the electrostatic interaction. Also, the exchange and polarization interactions play comparatively lesser but significant part in the stabilization. In the case of Cpd I–water complex, both electrostatic and polarization effects seem to constitute the main driving force.

## Supplementary Materials

Supplementary materials can be accessed at: http://www.mdpi.com/1420-3049/18/6/6782/s1.
