# Supplementary material for: Water Complexes of Cytochrome P450: Insights from Energy Decomposition Analysis
Source: Molecules. 2013 Jun 10;18(6):6782–91. doi: 10.3390/molecules18066782 (PMC6270449; doi:10.3390/molecules18066782)
Supplement: Supplementary file 1 [file molecules-18-06782-s001.pdf]

Supplementary Materials

XYZ coordinates of optimized geometries

Resting state (**1**)

H	10.73533440	33.60361862	1.92623603
S	9.73694611	32.81611633	2.38753700
Fe	10.50351238	30.86384010	1.62658095
N	10.94798470	31.59665298	-0.18784700
N	12.41124439	31.22996330	2.19746804
N	10.13799000	29.92940331	3.39059591
N	8.67722702	30.30350685	1.00315797
C	10.07916260	31.70023346	-1.24495697
C	10.72639275	32.35028076	-2.36046195
C	11.99533844	32.64094543	-1.96484196
C	12.12419891	32.17109680	-0.60600001
C	13.39723110	31.87138748	1.48016798
C	14.57034683	32.05105209	2.29733300
C	14.28213692	31.52644348	3.52145696
C	12.93334770	31.02113724	3.45497108
C	10.97835541	29.90678215	4.48099995
C	10.32184219	29.27675247	5.60008001
C	9.07286072	28.92896652	5.18205118
C	8.96135044	29.34908485	3.80780911
C	7.69866991	29.65620232	1.71698201
C	6.50360107	29.51994133	0.91784501
C	6.76853991	30.10012627	-0.28345099
C	8.12661171	30.58883667	-0.22049101
C	8.77002621	31.23806190	-1.26520598
H	8.20347691	31.39175797	-2.17859292
C	13.27249527	32.30047607	0.16472700
H	14.13002968	32.78843307	-0.28826699
C	12.27427387	30.40625954	4.51267385
H	12.81818962	30.31369972	5.44790411
C	7.82397985	29.20394897	3.02413702
H	6.96250296	28.71927643	3.47302794
H	12.78325272	33.12716293	-2.52607203
H	10.25588322	32.54740906	-3.31539392
H	15.48630047	32.52355576	1.96560299
H	14.91406059	31.48006439	4.39947510
H	10.77069092	29.12979126	6.57437801
H	8.28656292	28.43930435	5.74256992
H	5.59127092	29.04128075	1.25050199
H	6.11922789	30.19614220	-1.14446604
O	11.25768757	28.91538620	0.96324402
H	10.96628475	28.39050293	1.73022103
H	12.20640469	29.07636070	1.11366904

Cpd I•••H_2O (**2**)

H	1.65500498	-0.10018000	2.49330401
S	0.32352501	-0.31982300	2.40528607
Fe	0.08299500	0.00973500	-0.12181800
N	1.27486098	-1.62789905	-0.05936700
N	-1.52521598	-1.17739606	0.15897600
N	-1.07774997	1.63100195	0.12460600
N	1.72421503	1.18269897	-0.11085200
C	2.64564204	-1.65714395	-0.18979099
C	3.11361003	-3.02046299	-0.23882900
C	2.01407290	-3.81702209	-0.15928499
C	0.87096798	-2.94361997	-0.05766100
C	-1.55532300	-2.54879904	0.13125800
C	-2.91199803	-3.02335691	0.25375599
C	-3.70417690	-1.92200994	0.35865900
C	-2.83169508	-0.77601802	0.29130501
C	-2.43805504	1.65940595	0.26375800
C	-2.91170406	3.02009296	0.32567099
C	-1.81533897	3.81863809	0.21956401

C	-0.67714202	2.94102407	0.09579400
C	1.75135398	2.55336189	-0.11771500
C	3.10699010	3.02902293	-0.24347900
C	3.90106106	1.92626202	-0.32229900
C	3.02701211	0.78157002	-0.24189501
C	3.46055102	-0.54030102	-0.27920100
H	4.52749014	-0.71005499	-0.38765001
C	-0.44378501	-3.37414098	0.01657800
H	-0.61882401	-4.44550085	0.00096000
C	-3.25990200	0.54127502	0.34640300
H	-4.32635307	0.71477598	0.44843099
C	0.63697100	3.37571812	-0.01709600
H	0.80671901	4.44803476	-0.02957500
H	1.96226001	-4.89815903	-0.16849799
H	4.15242481	-3.31114006	-0.32878199
H	-3.20035911	-4.06662607	0.26480001
H	-4.77957106	-1.87106097	0.46998200
H	-3.94976592	3.30648398	0.43303800
H	-1.76265597	4.89967585	0.22467600
H	3.39472890	4.07222223	-0.26721099
H	4.97746420	1.87427104	-0.42394301
O	-0.02538800	-0.00281600	-1.74891198
H	-1.66822898	0.43125701	-2.61780405
O	-2.58549690	0.38581800	-2.95156407
H	-2.69472194	-0.55867499	-3.13638306

Water dimer (**3**)

O	-0.014038	0.177981	0.088324
H	0.239554	-0.111539	0.979257
H	0.774446	0.005137	-0.450548
O	-1.248545	-2.299366	-0.642059
H	-0.973971	-1.386442	-0.428362
H	-2.139855	-2.198896	-1.005662
